# Rehydration Driven Acid Impregnation of Thermally Pretreated Ca-Bentonite—Evolution of the Clay Structure

**DOI:** 10.3390/ma15062067

**Published:** 2022-03-11

**Authors:** Krzysztof Bahranowski, Agnieszka Klimek, Adam Gaweł, Zbigniew Olejniczak, Ewa M. Serwicka

**Affiliations:** 1 Faculty of Geology, Geophysics and Environmental Protection, AGH University of Science and Technology, al. Mickiewicza 30, 30-059 Krakow, Poland; aklimek@agh.edu.pl (A.K.); agawel@agh.edu.pl (A.G.); 2 Institute of Nuclear Physics, Polish Academy of Sciences, Radzikowskiego 152, 31-342 Krakow, Poland; zbigniew.olejniczak@ifj.edu.pl; 3 Jerzy Haber Institute of Catalysis and Surface Chemistry, Polish Academy of Sciences, Niezapominajek 8, 30-239 Krakow, Poland; ncserwic@cyf-kr.edu.pl

**Keywords:** bentonite, thermal activation, acid-activation, dry impregnation

## Abstract

A new approach to acid activation of raw Ca-bentonite was explored. The method consisted in dehydration of clay by thermal pretreatment at 200 °C, followed by immediate impregnation with H_2_SO_4_ solution. The acid concentration was 1.5 × or 2.0 × cation exchange capacity (CEC) of clay. The volume of the liquid was adjusted so as to leave the material in the apparently dry state. Structural evolution of the activated solids after 1, 2, 3, and 4 weeks of storage was monitored with X-ray diffraction (XRD), Fourier transform infrared spectroscopy (FTIR), ^27^Al magic angle spinning nuclear magnetic resonance (MAS NMR), and chemical analysis. In the macroscopically dry solids, the rehydrated interlayer Ca^2+^ underwent rapid exchange with H_3_O^+^ and formed extra-framework gypsum. Acid attack on montmorillonite structure resulted in continuous removal of layer forming Mg, Al, and Fe cations, with Mg^2+^ being eliminated most efficiently. No significant damage to the montmorillonite lattice was observed. Al was extracted both from the tetrahedral and the octahedral sheets. Under less acidic conditions, the monohydrated H-montmorillonite changed upon storage to bi-hydrated form, as a result of clay auto-transformation. Higher concentrations of acid in the pore network of clay stabilized the H-form of montmorillonite. The data indicate that compositional transformation of acid impregnated bentonite extended beyond the one month of aging investigated in the present work.

## 1. Introduction

Transformation of clay minerals upon contact with an acidic environment may occur in nature, as a result of common geological weathering processes, or as an undesired side effect of human activities, e.g., mining (due to the drainage of acidic waste waters into the ecosystem) [1,2]. However, the process referred to as acid activation is understood as purposeful chemical treatment of clays, widely used in the industry for manufacturing of clay-derived functional materials, such as bleaching earths (decolorizing agents), sorbents, or catalysts [3]. Although the literature reports describe acid treatment of different kinds of clays, the bulk of research, as well as industrial interests, concentrate on acid treatment of bentonites, the montmorillonite-bearing rocks. Montmorillonite is a phyllosilicate mineral, whose basic building unit is a layer composed of octahedral Al-based sheet sandwiched between two tetrahedral Si-based sheets. Due to the partial substitution of Al^3+^ with Mg^2+^ in the octahedral sheet, and also a degree of substitution of Si^4+^ with Al^3+^ in the tetrahedral sheet, the layers are negatively charged, which is compensated by the presence of cations in the interlayer, predominantly Na^+^ and/or Ca^2+^. The interlayer also contains water molecules. 

The discovery that bentonite can develop enhanced decolorizing ability by treatment with acid was made at the beginning of the last century in Germany [4]. Industrial production of acid activated bentonite for bleaching purposes began there already in 1909. In particular, the brand name Tonsil, introduced at that time, continues to be used by the successive manufacturers (currently Clariant) until now, and is frequently considered a synonym for activated bleaching earth. As an example of the current application, the widespread use of acid activated bentonite for decolorization/purification of vegetable, animal, and mineral oils can be mentioned. Over time, the scope of applications of the acid activated clays broadened beyond the original use as decolorizing agents, to include adsorption of other undesired substances, exploitation in catalysis and photocatalysis, or use of the ion exchange properties, the aspects addressed in a number of reviews [3,5,6,7,8,9,10,11,12,13,14,15,16]. Of growing importance is the potential of acid activated clays in the design of adsorbents and/or catalysts capable of removal/destruction of toxic substances polluting the environment. Accordingly, many variations of preparative approaches to acid treatment of bentonites, depending on the purpose of modification, have been developed and reported in the literature in the course of the years. Most frequently activation is carried out with sulfuric or hydrochloric acids, the properties of the final product depending chiefly on the acid concentration, treatment temperature and time, acid/clay ratio, or the extent of washing [17,18,19,20,21,22,23,24,25,26,27,28,29,30]. In the procedure referred to as impregnation, the liquid phase is removed by evaporation and the washing step eliminated, so that the resulting solids may be considered as acids supported on the clay mineral [20]. The diversity of the preparative approaches to acid treatment of bentonites for various purposes is illustrated by selected examples listed in Table 1. 

The acid treatment of bentonite proposed in the present work is a novel variation of an impregnation procedure, taking advantage of the known ability of a dehydrated montmorillonite to spontaneously rehydrate and swell upon contact with water [31,32,33]. The approach involves a dehydrating thermal pretreatment of bentonite, at temperature ensuring removal of interlayer water. The hot dry product is immediately treated with aqueous solution of H_2_SO_4_, which acts simultaneously as a medium enacting rehydration of interlayer cations and a carrier of protons penetrating the clay structure. We have recently successfully employed this concept to accomplish an efficient Na-activation of bentonite with soda solution [34]. Noteworthy, the volume of acid is attuned in such a way as to effectuate a dry impregnation, i.e., after treatment with acid solution, the activated clay remains in an apparently dry state, ready for the potential use. The present study aims at providing insight into the evolution of the clay structure upon the applied acid treatment.

## 2. Materials and Methods

A calcium-rich bentonite from Kopernica deposit in Slovakia (supplied by CERTECH, Niedomice, Poland), containing ca. 90% of montmorillonite, was used in the experiments. Recently published detailed mineralogical characterization of this bentonite [35] showed that montmorillonite was the only smectite mineral present in the raw material. Its abundance and the nature of accessory minerals depended on the location of the mining site. Physicochemical characterization revealed that in addition to Al present in the octahedral sheet, a degree of Al for Si substitution in the tetrahedral sites occurred. Prior to the acid treatment the raw clay was homogenized by blending in a ceramic mortar and oven dried (BMT Medical Technology, Brno, Czech Republic) for 3 h at 200 °C. The determination of the pretreatment temperature was based on the results of thermal analysis of the Kopernica bentonite [34] and on the previous work indicating 200 °C as the temperature at which interlayer water can be eliminated from Ca-montmorillonite, without starting the dehydroxylation process [36,37]. Straight after taking out from the oven, 100 g of the dried bentonite was subjected to impregnation with appropriately diluted concentrated H_2_SO_4_ (98%, Chempur, Piekary Slaskie, Poland) under vigorous stirring. Two samples were prepared by using H_2_SO_4_ solutions of concentration corresponding to 1.5 × and 2.0 × CEC of bentonite, prepared by diluting 3.2 and 4.2 mL of concentrated acid in 35 mL of distilled water (14% and 18% acid solutions), respectively. The resulting acid/clay ratio was ca. 0.4 mL/g. The volume of water used during impregnation was determined as the maximum amount leaving the sample in the apparently dry state. Structural characterization of the samples was performed immediately after activation and following 1, 2, 3, and 4 weeks of storage, after which the XRD pattern did not change. The non-treated bentonite is referred to as sample N, while the samples activated with acid corresponding to 1.5 × and 2.0 × CEC are denoted 1.5/n and 2.0/n, where n = 0 refers to the freshly impregnated sample, and n equal 1, 2, 3, or 4 describes the time of storage (in weeks). 

Powder X-ray diffraction (XRD) patterns of pressed powder samples were recorded with a Rigaku SmartLab diffractometer (Rigaku, Tokyo, Japan), at 50% relative humidity (RH). Graphite-monochromatized CuKα radiation, operating voltage = 45 kV, current = 200 mA, 2θ step size = 0.05° and counting time = 1 s/step were used.

Fourier transform infrared (FTIR) absorption spectra were recorded in middle infrared (MIR), in the 4000–400 cm^−1^ range, using a Nicolet 6700 spectrometer (Thermo Scientific, Madison, WI, USA). 2 mg of bentonite powder was mixed with 200 mg of KBr model, (Sigma-Aldrich, Poznan, Poland) and pressed into a pellet with 10 MPa pressure. For each spectrum 64 scans at 2 cm^−1^ resolution were accumulated. 

High-resolution, solid-state ^27^Al magic angle spinning nuclear magnetic resonance (MAS NMR) spectra were recorded with the APOLLO console (Tecmag Inc., Houston, TX, USA) and the 7 T/89 mm superconducting magnet (Magnex Scientific, Abingdon, UK). A Bruker HP-WB high speed MAS probe equipped with the 4 mm zirconia rotor and the KEL-F cap was used to spin the sample at 8 kHz. The resonance frequency was 78.068 MHz. A single 2 μs rf pulse, corresponding to π/6 flip angle in the liquid, was used. The acquisition delay in accumulation was 1 s, and 1000 scans were acquired. The ppm scale was referenced to 1 M solution of Al(NO_3_)_3_ (Chempur, Piekary Slaskie, Poand). 

The cation exchange capacity (CEC) of the starting bentonite was established with aid of the BaCl_2_ replacement method [38]. Briefly, 10 mL of 0.1 M BaCl_2_ was added to 0.2 g of the clay sample, treated for 5 min in the ultrasonic cleaner Sonic 2 (Polsonic, Warsaw, Poland) and mixed in a rotator (JW Electronic, Warsaw, Poland) for 2 h. The sample was centrifuged, and the supernatant collected. The solid residue was washed five times by centrifugation, all supernatants blended together and subjected to analysis of exchanged cations (Ca, Na, K) by atomic absorption spectrometry method, using Thermo Scientific 3500 (Thermo Electron Manufacturing, Cambridge, UK) spectrometer. The BaCl_2_ replacement method was also used to determine the amount of Mg, Fe, and Al removed from the clay structure upon impregnation with acid. 

## 3. Results and Discussion

### 3.1. XRD Analysis of Acid Activated Bentonites

**Figure 1:**
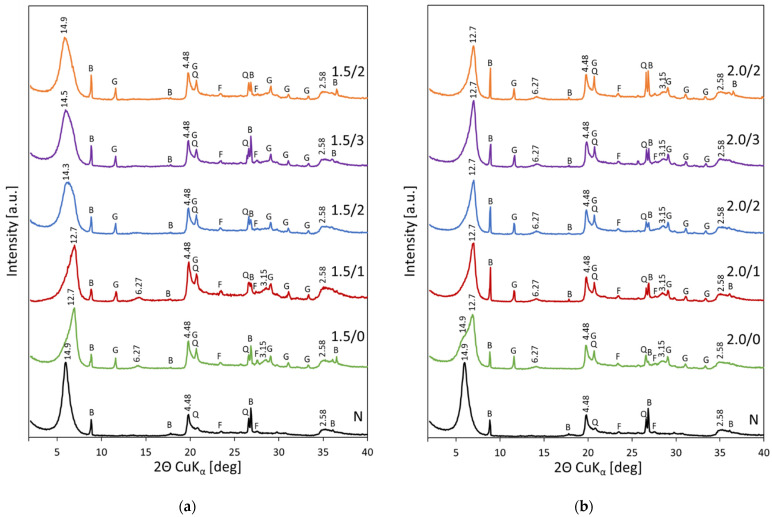
Powder XRD patterns of bentonite activated with H_2_SO_4_ in quantities corresponding to: (**a**) 1.5 × CEC; (**b**) 2.0 × CEC; G—gypsum, B—biotite, Q—quartz, F—feldspar.

**Figure 2:**
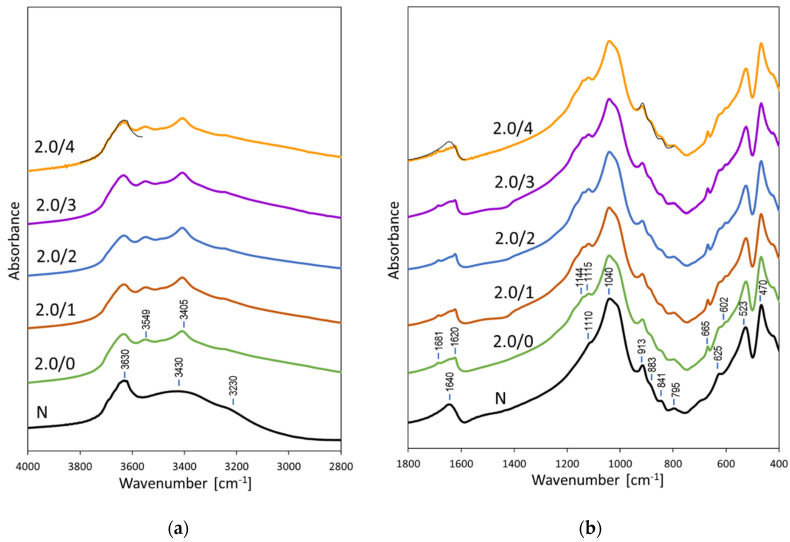
Evolution with the time of aging of FTIR patterns of bentonite impregnated with H_2_SO_4_ in quantity corresponding to 2.0 × CEC of clay: (**a**) 2800–4000 cm^−1^ range; (**b**) 400–1800 cm^−1^ range. To facilitate comparison the relevant fragments of spectrum of non-treated bentonite are superimposed on the spectrum of 2.0/4 sample (thin black line trace).

Powder XRD patterns of bentonite subjected to acid treatment with H_2_SO_4_ solution of concentrations corresponding to 1.5 × and 2.0 × CEC of clay are presented in Figure 1a,b, respectively. Diffractograms illustrate the evolution of the XRD patterns with the time of storage. The non-treated bentonite shows the *d*_001_ value equal 14.9 Å, as expected for the Ca-form of montmorillonite, in which the Ca^2+^ cations are encased in the double layer of water molecules. The peaks with *d* value of 4.48 and 2.58 Å are related to the 100 and 110 reflections of montmorillonite. Reflections of the minority mineral components of the Kopernica bentonite, such as feldspar, mica and quartz [35], are also visible. Upon treatment with H_2_SO_4_ solution of concentration equivalent to 1.5 × CEC an immediate change in the profile of 001 reflection is observed (Figure 2a). The maximum shifts to 12.7 Å, and the remnants of the 14.9 Å reflection are visible as the low angle peak asymmetry. The appearance of 12.7 Å maximum indicates that bulk of the smectite component contains single sheet of water in the interlayer [39]. In the RH conditions of the XRD measurement carried out in the present study, such value points to the formation of H-form of montmorillonite and confirms that a replacement of most of the Ca^2+^ cations with hydronium ions occurred. The change from bi-hydrated to monohydrated state upon replacement of Ca^2+^ with H_3_O^+^, is consistent with the findings of Ferrage et al. [40] who studied the effect of acidification on the interlayer cationic composition and hydration state of Ca-montmorillonite. It is further validated by the appearance, in the acid treated materials, of reflections characteristic of CaSO_4_ dihydrate (gypsum) precipitate, with the dominant one at 2Θ = 11.6° (*d*_020_ = 7.63 Å). Kopernica bentonite does not contain Ca-bearing impurities (such as, e.g., calcite) that might yield CaSO_4_ upon reaction with H_2_SO_4_; therefore, the observed formation of gypsum must be caused by the reaction of Ca^2+^ ions expelled from the interlayer with the sulfate anions available in the clay pore network. The amount of precipitated CaSO_4_ dihydrate is similar in all recorded patterns, which shows that the exchange of interlayer Ca^2+^ cations with hydronium ions occurs very fast. It should be noted that the precipitation of extra-framework gypsum acts as an additional driving force of H_3_O^+^ for Ca^2+^ exchange (beside the ion concentration gradient).

Upon aging, the XRD pattern of acid activated bentonite undergoes further change, as, gradually, the maximum of the basal reflection moves back to the lower 2Θ values. After 4 weeks of storage the *d*_001_ value again equals 14.9 Å, i.e., assumes position characteristic of montmorillonite with double sheet of water in the interlayer. The effect points to substantial change in the nature of compensating cations, which may be attributed to the so-called autotransformation of proton-saturated smectites, known to occur during aging [13]. In parallel to the swift exchange of interlayer metal cations with hydronium species, the acid treatment results in leaching of structural layer-forming cations. The acid attack proceeds primarily from the layer edges but leaching via the interlayer plays an important role as well [13,41]. The latter path involves migration of protons from the interlayer into the layers and attack on the structural OH groups, preferentially those associated with sites responsible for the deficit of the positive layer charge (Mg^2+^ or Fe^2+^ substituting for Al^3+^ in the octahedral sheet and Al^3+^ substituting for Si^4+^ in the tetrahedral sheet). This leads, eventually, to the release of layer Al, Fe, and Mg cations and their migration to the interlayer. As a result, a spontaneous transformation of H-smectites to their Al-, Fe-, or Mg- forms occurs. In view of this, the observed shift of the *d*_001_ value from 12.7 Å, characteristic of mono-hydrated H-montmorillonite, to 14.9 Å, indicative of bi-hydrated state typical for Al, Mg, or Fe interlayer cations, is consistent with the replacement of the hydronium ions with cationic species extracted from the layers during autotransformation.

It should be noted that the mono-hydrated H-form of montmorillonite appears well ordered in the *c* direction, as higher order 002 and 004 reflections, corresponding to *d* values equal 6.27 and 3.15 Å, respectively, become visible in the XRD pattern. The intensity of higher order reflections in smectite is strongly dependent on the organization of cations and water molecules in the interlayer [39]. In bi-hydrated forms of montmorillonite, with different interlayer packing of higher valent cations, the higher order reflections are of much lower intensity than the ones in smectite with single water sheet. For this reason, neither the Ca-form, nor the auto-transformed montmorillonite with Al, Fe, and/or Mg cations in the interlayer show the higher 001 reflections.

Evolution of XRD pattern of bentonite impregnated with acid of higher concentration, corresponding to 2.0 × CEC of clay, is shown in Figure 1b. The changes in the spectra recorded immediately after treatment and after one week of storage are similar to those observed for impregnation with less concentrated acid. Initially the (001) maximum shifts to higher 2Θ and assumes position characteristic of H-montmorillonite with *d*_001_ = 12.7 Å, with a only a weak shoulder around 15 Å, attributable to the remainder of bi-hydrated clay areas. After one week the 12.7 Å maximum is the only feature in the area of basal reflection, indicating that the H_3_O^+^ for Ca^2+^ exchange resulted in monohydrated interlayers throughout the sample. Extending the aging period for over one week brings no further changes to the XRD patterns of bentonite. In particular, there is no increase of the basal spacing that might point to the interlayer becoming populated by Al, Mg, or Fe cations extracted from the layers in the course of autotransformation. Apparently, the large excess of sulfuric acid present in the pore system of bentonite ensures a rapid replacement of cations released to the interlayer with incoming hydronium species, so that the monohydrated state is retained.

Many works on the wet acid activation of bentonites reported that intensification of the treatment, e.g., by increase of acid concentration or by prolongation of the exposure time resulted eventually in worsening of clay mineral crystallinity [18,19,23,24,25,28,29,42,43,44,45,46]. Analysis of the XRD patterns in Figure 1 shows that the employed dry acid impregnation of bentonite is a relatively mild treatment and does not induce any significant loss of long range order in the montmorillonite component. This is especially visible in Figure 1b, showing the structural evolution of bentonite upon impregnation with more concentrated acid. The H-montmorillonite generated during this treatment retains the line shape of its basal reflection as well as the higher order 002 and 004 reflections in an essentially unchanged form, irrespective of the time of aging. The mildness of the method may be considered an advantage, as it enables facile formation of H-montmorillonite without affecting the essential features of the montmorillonite structure.

### 3.2. FTIR Analysis of Acid Activated Bentonites

Spectroscopy in the mid-infrared region enables insight into the short-range structural evolution of acid-treated clay minerals, because bands characteristic of bonding within the layers, especially those characteristic of vibrations involving OH groups and/or octahedral cations, are sensitive to changes induced by proton attack [3,21,47,48,49,50]. FTIR spectra of Kopernica bentonite impregnated with H_2_SO_4_ solutions of the concentration corresponding to 2.0 CEC× of clay are shown in Figure 2a, which displays the 2800–4000 cm^−1^ range where OH stretching vibrations appear, and Figure 2b, which shows the 400–1800 cm^−1^ area, where water bending modes and lattice vibrations can be found. The spectra of bentonite straight after activation and after storage for 1, 2, 3, and 4 weeks are compared with the spectrum of non-treated clay. The latter is typical of raw bentonite rich in montmorillonite [50,51], with the 3630 cm^−1^ band due to stretching vibrations of OH groups bonded to octahedral cations in Al_2_OH, AlMgOH, and AlFeOH ensembles, the broad band around 3430 cm^−1^ stemming from stretches of OH groups in hydrogen bonded water molecules, and the shoulder at 3230 cm^−1^ associated with the overtone of H_2_O bending vibration at 1640 cm^−1^. The lattice vibrations below 1200 cm^−1^ are dominated by the maximum at 1040 cm^−1^ related to the in-plane Si-O-Si stretches. The shoulder at 1110 cm^−1^ stems from out-of-plane Si-O vibrations. The features visible in the OH bending region (950–800 cm^−1^), i.e., the bands at 913 cm^–1^, 883 cm^−1^, and 841 cm^−1^, are due to Al_2_OH, AlFeOH, and AlMgOH bending modes, respectively. The 795 cm^–1^ band is due to the presence of quartz impurities, the 625 cm^–1^ band arises from the coupled Al-O out-of-plane and Si-O modes, and the bands at 523 and 470 cm^–1^ originate from Al_oct_–O–Si and Si–O–Si bending vibrations, respectively.

In FTIR spectra of H_2_SO_4_ impregnated bentonite, next to the features characteristic of montmorillonite, a set of new bands appear, unequivocally pointing to the formation of CaSO_4_∙2H_2_O deposit. The 3549, 3405 cm^−1^ bands are due to the stretching vibrations of hydroxyls in structural water molecules of gypsum, the 1681, 1620 cm^−1^ doublet originates from two coupled H_2_O bending vibrations, and the bands at 1144, 1115 cm^−1^, and 665, 602 cm^−1^, correspond to the stretching and bending vibrations of sulfates, respectively [52]. In consistence with the XRD data, FTIR spectra point to precipitation of gypsum straight after acid impregnation. Further storage does not change the gypsum bands intensity, which confirms that cation exchange of Ca^2+^ with hydronium ions and precipitation of the extra-framework CaSO_4_∙2H_2_O is a rapid process. The bands belonging to montmorillonite appear almost unchanged, thus corroborating the conclusion as to the mildness of the applied acid treatment drawn on the basis of XRD analysis. However, careful inspection of the spectra in the ranges most likely to be affected by acid treatment reveals some small differences, best visible after superposition of the relevant fragments of the spectrum of non-treated bentonite on the spectrum of sample aged for 4 weeks (Figure 2a,b). Although the band at 3630 cm^−1^, associated with stretches of OH groups coordinated to octahedral Al, Mg and Fe cations remains practically unaffected, the set of bands due to Al_2_OH, AlFeOH and AlMgOH bending modes lose some of their intensity. This shows that while the degree of dehydroxylation resulting from proton attack on the layer hydroxyls did not change their concentration in a meaningful way, the associated extraction of cations from octahedral sheet left some mark on the spectrum. In addition, the lower intensity of water band at 1640 cm^−1^ reflects the change from the bi-hydrated to mono-hydrated interlayer occurring upon transformation from Ca- to H-form of montmorillonite.

### 3.3. Quantitative Assessment of Metal Cations Released from the Layers upon Acid Treatment

**Figure 3:**
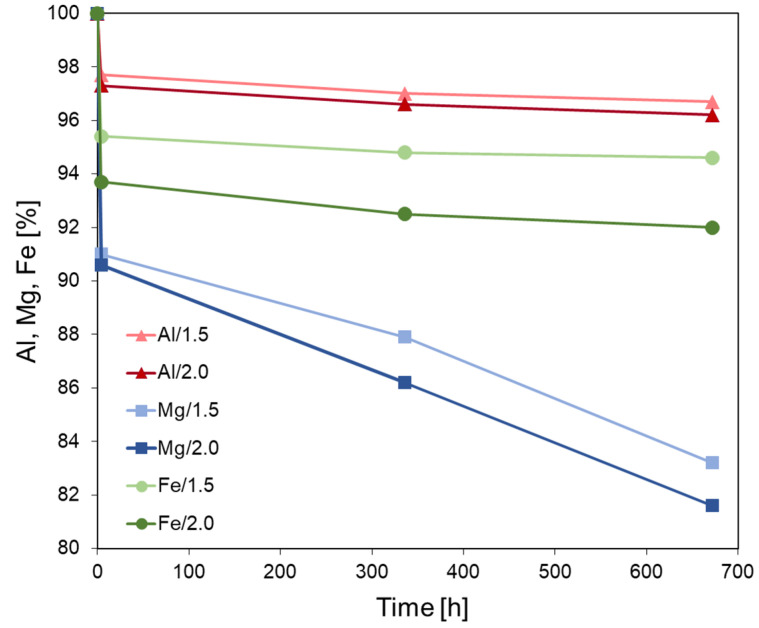
Evolution of Al, Mg, and Fe content in the clay structure with time after acid treatment.

In order to estimate the amount of Al, Mg, or Fe cations released from the structure of acid impregnated bentonites after different periods of aging, the BaCl_2_ replacement method was employed. Treatment with excess of BaCl_2_ aqueous solution enables removal of all exchangeable Al, Mg, or Fe cations that have been extracted from the layers either to the interlayer or to the extra-framework positions, where they exist as soluble amorphous sulfate deposits. The results of chemical analysis of the eluents are presented in Figure 3, which shows the undissolved % fraction of the respective cations.

It is evident that the loss of Al, Mg, or Fe starts immediately after acid impregnation and continues in the macroscopically dry sample over the whole investigated period. The use of more concentrated acid accelerates the metal cations leaching. Magnesium is removed more readily than Fe and Al, in agreement with previous reports on the effect of acid treatment [19,22,24,41,53]. A rapid loss of metal cations is observed at the very beginning of impregnation, followed by a slower release during aging. The initial effect is attributed primarily to the increased temperature at the beginning of impregnation. Firstly, the acid is poured over hot sample, taken out from the oven heated to 200 °C, secondly, rehydration of dehydrated interlayer cations is an exothermic process, causing an increase of clay temperature. Both factors must result in the rate of dissolution higher than that observed in the material cooled down to room temperature. Moreover, the loss of cations from easily accessible surface sites, more facile than their extraction from the depth of the clay layers which dominates the later stages of clay-acid interaction, may also contribute to the initial rapid dissolution. From the graphs in Figure 3, especially the steep fall of Mg content, it may be concluded that upon one months of storage the activation induced compositional transformation of clay has not yet been completed.

Comparison with the literature data show that the cation loss from the structure of bentonite dry impregnated with sulfuric acid is much lower than that observed for wet treatments of bentonites with hot H_2_SO_4_ solutions of comparable concentrations [21,22]. Thus, the results of the chemical analysis provide additional evidence of “soft” character of the presented method of acid activation.

**Figure 4:**
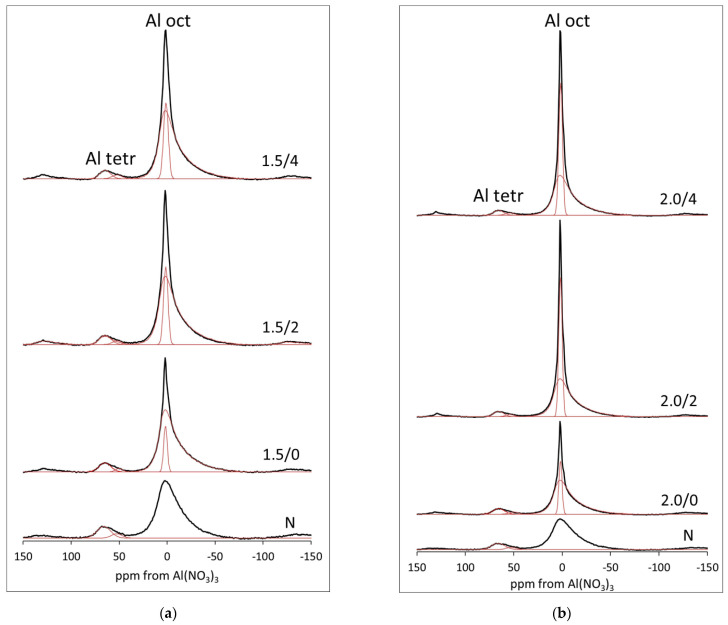
Evolution of ^27^Al MAS NMR spectra of bentonite impregnated with H_2_SO_4_ with time of aging: (**a**) series treated with H_2_SO_4_ of concentration corresponding to 1.5 × CEC (**b**) series treated with H_2_SO_4_ of concentration corresponding to 2.0 × CEC.

The chemical analysis data on the Al loss from the solid, presented in Figure 3, cover the effect caused jointly by leaching of Al from the octahedral sheet, where it constitutes the main structure-forming cation, and from the tetrahedral sheet, where it substitutes for Si and is a minority species. In order to get insight into the impact of acid treatment on each kind of structural Al, the selected samples from both series have been subjected to analysis with ^27^Al MAS NMR spectroscopy, well-suited to distinguish between Al species with different coordination spheres [54]. The obtained spectra are presented in Figure 4, and the parameters of spectral components derived from deconvolution are gathered in Table 2.

All ^27^Al MAS NMR spectra show two maxima, around 0 and 60 ppm, corresponding to Al in the octahedral and tetrahedral coordination, respectively, as expected for montmorillonite-rich bentonite with a degree of Al for Si substitution in the smectite mineral [17,35,47,55,56]. In the non-treated sample N, the main feature at ca. 0 ppm is a single wide resonance due to the structural Al in the octahedral sheet. On the other hand, the peak around 60 ppm is an envelope of two components (Figure 4), whose position and relative share in the spectrum are given in Table 2. The more intense one, at 67.5 ppm, is due to the tetrahedral Al in bi-dimensional silicate sheet of montmorillonite, the other, at 54.0 ppm, corresponds to Al in the three-dimensional Si-O framework, such as that of feldspar impurity. The major modification found in the spectra of samples treated with H_2_SO_4_ is the appearance, beside the wide resonance from octahedral layer Al, of a much narrower NMR signal pointing to the formation of another type of octahedral Al species. The sharpness of this peak indicates highly mobile Al form, such as hydrated Al cations with a degree of rotational freedom. Obviously, the species result from the acid hydrolysis of structural Al, and their contribution increases with the time of aging and with the concentration of H_2_SO_4_ used for impregnation. Rhodes and Brown [55] also reported narrow ^27^Al MAS NMR signals, characteristic of hydrated Al^3+^ cations with rotational freedom, in aged acid-treated samples. The mobile Al may be located either in the interlayer, as compensating cations, or in the extra-framework positions of the acid impregnated clay. However, it should be remembered that the degree of hydration of non-framework Al is very labile. Upon dehydration, the coordination sphere of Al loses symmetry, which eventually leads to very broad, undetectable NMR signals [57]. Moreover, some of the Al sites remaining in the structure damaged by the acid attack may display lower symmetry and also become invisible. Nevertheless, although ^27^Al MAS NMR may not reveal all Al centers present in the analyzed material, the results show clearly that Al is extracted both from the octahedral and from the tetrahedral sheets, and that at least part of the leached Al species form mobile, solvated cations.

**Figure 5:**
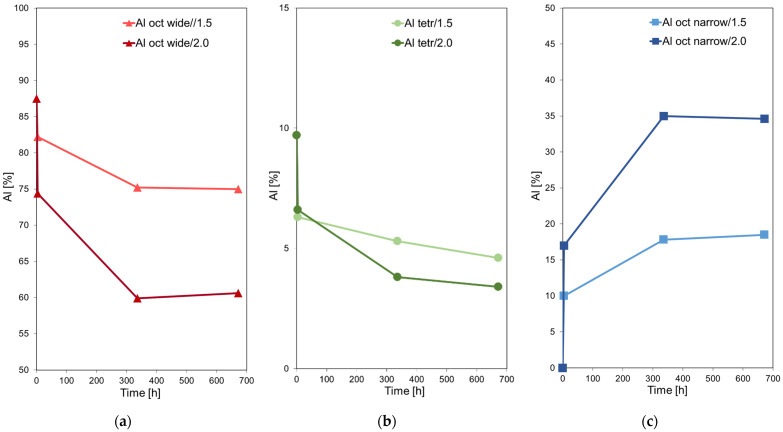
Evolution with time of aging of various montmorillonite-related Al species detected by ^27^Al MAS NMR: (**a**) wide component of the octahedral Al corresponding to lattice Al; (**b**) tetrahedral Al component corresponding to Al substituting for Si in the silica sheet; (**c**) narrow component of the octahedral Al, corresponding to Al extracted from the layers.

Graphical illustration of the evolution of various montmorillonite-related Al species with time of aging is presented in Figure 5. It is apparent that the most pronounced loss of both the octahedral and the tetrahedral Al from the structure occurs immediately after impregnation with acid, followed by a slower release upon aging, in agreement with the kinetic data obtained by the chemical analysis.

## 4. Conclusions

The proposed approach to acid activation of Ca-bentonite, consisting in a dehydration of clay at 200 °C, followed by rehydration driven impregnation with aqueous solution of H_2_SO_4_, enables facile preparation of acid-activated clay in the dry state. In comparison with conventional wet activation, the approach limits the number of operational stages, making the overall process easier and faster. Dry acid impregnation described in this work may be considered a relatively mild treatment, as it does not induce any significant destruction of the montmorillonite lattice. The observed structural evolution is essentially limited to exchange/migration of interlayer and lattice-forming cations, with only weak dehydroxylation of the clay mineral layers. Physicochemical characterization of the solids by combined use of chemical analysis, XRD, FTIR, and ^27^Al MAS NMR techniques revealed that in the macroscopically dry solids the rehydrated interlayer cations underwent rapid exchange with hydronium ions. The process was accompanied by the precipitation of extra-framework gypsum. Attack of protons on the layers was a slower process, which continued over the whole investigated aging period. The acid attack on the montmorillonite structure resulted in the continuous removal of layer forming Mg, Al, and Fe cations, with Mg^2+^ being eliminated most efficiently. Al was extracted both from the tetrahedral and the octahedral sheets. Under less acidic conditions the monohydrated H-montmorillonite changed upon storage to bi-hydrated form, the effect interpreted as due to migration of leached Mg, Al, and Fe cations to the interlayer, in the process of clay mineral autotransformation. The occurrence of auto-transformation indicated that the acid attack proceeded not only from the edges, but also from the interlayer. Higher concentration of acid in the pore network of clay stabilized the H-form of montmorillonite. The data indicate that the compositional transformation of acid impregnated bentonite extends beyond the one month of aging time investigated in the present work.

## Figures and Tables

**Table 1:** Examples of acid-activation procedures described in the open literature (Bent—bentonite, Mt—montmorillonite).

Activation Procedure	Purpose	Reference
Mt + 6 M HCl, acid/clay ratio 200 mL/g, 95 °C, 1–24 h, washed with water and dried at 60 °C.	Study of acid dissolution by ^27^Al and ^29^Si MAS NMR	[17]
Bent + H_2_SO_4_ (0.5–4 M), acid/clay ratio 5 mL/g, 80 °C, 2 h, washed with water.	Study of textural properties and surface acidity	[18]
Bent + HCl (0.5–8 M), 70 °C, acid/clay ratio 15 mL/g, 30 min–6 h, washed with water and dried at 100 °C.	Optimization of bleaching properties	[19]
Bent + 0.5 M H_2_SO_4_, acid/clay ratio 5 mL/g, H_2_O removed by evaporation, dried at 100 °C, calcined at 300 °C, 2 h.	Catalyst design	[20]
Mt + H_2_SO_4_ (0.5–5 M), 80 °C, 4 h, washed with water and dried at RT + 6 h at 120 °C.	Study of structural evolution	[21]
Bent + H_2_SO_4_ (3, 4, or 5 M), acid/clay ratio 10 mL/g, 30–90 °C, 15–120 min.	Study of activation kinetics	[22]
Bent + H_2_SO_4_ (0–70 wt% of the mixture), acid/clay ratio 20 mL/g, 97 °C, 6 h, washed and dried 4 h at 105 °C.	Study of structural, compositional and textural evolution	[23]
Bent + H_2_SO_4_ or HCl (1, 5, or 10 M), acid/clay ratio 100 mL/g, 80 °C, 1.5–96 h, washed with water and freeze-dried.	Study of structural evolution and dissolution kinetics	[24]
Bent + 2 M HCl, acid/clay ratio 7 mL/g, microwave heated to 100 °C for 1–20 min, washed with water and freeze-dried.	Study of textural properties	[25]
Bent + HCl (0.05–0.5 M), acid/clay ratio 0.1 mL/g, 60–100 °C, washed with water, dried 12 h at 55 °C.	Removal of dyes from waste water	[26]
Mt + 3.2 M HNO_3_, acid/clay ratio 49 mL/g, 104 °C, 4–24 h, washed with water, dried and calcined at 450–1150 °C for 4 h.	Catalyst design	[27]
Bent + HNO_3_ (1, 2, 4, or 8 M), 20–90 °C, 1–12 h, washed with water, dried 12–48 h at 60 °C.	Study of properties relevant for radioactive waste barriers	[28]
Mt + 3 M HCl, acid/clay ratio 15 mL/g, 95 °C, 1–24 h, washed with water, dried at 80 °C.	CO_2_ sorption	[29]
Bent + 5 M mixtures of H_2_SO_4_/HNO_3_, HNO_3_/H_3_PO_4_, or H_3_PO_4_/H_2_SO_4_, acid/clay ratio 10 mL/g, RT, 4 h, washed with water, dried at 60 °C.	Control of tap water conductivity	[30]

**Table 2:** Parameters of ^27^Al MAS NMR spectra components obtained from deconvolution.

Sample	^29^Si MAS NMR Parameter	Tetrahedral Al		Octahedral Al	
N	center (ppm)	67.5	54.0	2.9	-
	intensity contribution (%)	9.7	2.8	87.5	-
1.5/0	center (ppm)	66.0	54.7	2.9	2.1
	intensity contribution (%)	6.3	1.5	82.2	10.0
1.5/2	center (ppm)	66.2	53.5	2.9	2.1
	intensity contribution (%)	5.3	1.7	75.2	17.8
1.5/4	center (ppm)	66.1	53.6	2.9	2.1
	intensity contribution (%)	4.6	1.9	75.0	18.5
2.0/0	center (ppm)	66.9	54.5	2.9	2.1
	intensity contribution (%)	6.6	2.0	74.4	17.0
2.0/2	center (ppm)	67.2	55.5	2.9	2.1
	intensity contribution (%)	3.8	1.3	59.9	35.0
2.0/4	center (ppm)	67.2	55.6	2.9	2.1
	intensity contribution (%)	3.4	1.4	60.6	34.6

## Data Availability

The data presented in this study are available upon request from the corresponding author.
